# Triple Renal Arteries in a Cadaveric Kidney Donor: A Case Report

**DOI:** 10.7759/cureus.11639

**Published:** 2020-11-23

**Authors:** Christos Damaskos, Vasiliki E Georgakopoulou, Nikolaos Garmpis, Anna Garmpi, Dimitrios Dimitroulis

**Affiliations:** 1 Renal Transplantation Unit, Laiko General Hospital, N.S. Christeas Laboratory of Experimental Surgery and Surgical Research, Medical School, National and Kapodistrian University of Athens, Athens, GRC; 2 Pulmonology Department, Laiko General Hospital, Athens, GRC; 3 First Pulmonology Department, Sismanogleio Hospital, Athens, GRC; 4 Second Department of Propedeutic Surgery, Laiko General Hospital, Medical School, National and Kapodistrian University of Athens, Athens, GRC; 5 First Department of Propedeutic Internal Medicine, Laiko General Hospital, Medical School, National and Kapodistrian University of Athens, Athens, GRC; 6 Second Department of Propaedeutic Surgery, Laiko Hospital, Athens Medical School, National and Kapodistrian University of Athens, Athens, GRC; 7 Hellenic Minimally Invasive and Robotic Surgery (MIRS) Study Group, Athens Medical School, National and Kapodistrian University of Athens, Athens, GRC

**Keywords:** renal transplantation, renal artery, renal vasculature

## Abstract

Variation in the number of renal arteries is rare and is the most frequent and clinically important variation in the renal vascular system. Typically, this variant represents an immature form of complicated development of the renal arteries resulting from the persistence of more than one mesonephric artery during the transition period from mesonephros to metanephros in embryogenesis. The knowledge of this anatomical variation will allow the best healthcare to be provided for patients undergoing kidney surgical procedures and may reduce or eliminate avoidable postoperative complications. Although a double renal artery consists of a common anatomical variation, three or more arteries in a single kidney is less common. Herein, we report a case of a 42-year-old healthy cadaveric donor whose left kidney was found to have three renal arteries.

## Introduction

Normally, each kidney is supplied by a single renal artery (RA) arising from the abdominal aorta. Anatomical variations in the renal vascular system are described with an incidence of 20%-75% [1]. The most frequent variation is the presence of multiple RAs [2]. Accessory renal arteries present bilaterally in 10%-15% of cases while double renal arteries occur in an incidence of 20% (range 14%-23%) and triple renal arteries occur in an incidence of 2.5% (range 1%-4%) [3]. During the fetal period, multiple mesonephric arteries deliver blood to the kidney. Typically, one mesonephric artery persists and becomes the RA while the persistence of multiple mesonephric arteries can result in multiple RAs [4].

Although patients with multiple RAs typically are asymptomatic, knowledge of anatomical variations in the renal vascular system has diagnostic and therapeutic implications and is crucial for surgical procedures such as uroradiological procedures, vascular reconstruction, renal trauma, and kidney transplantation [5]. Multiple RAs can be detected through surgical procedures, cadaver dissection, or radiological examinations such as angiography, computed tomography (CT), ultrasonography, and magnetic resonance imaging (MRI) [6].

Our aim is to report our experience via a case of a cadaveric kidney donor, which, during the preparation of the kidney for transplantation, was found to have three RAs in his left kidney.

## Case presentation

**Figure 1:**
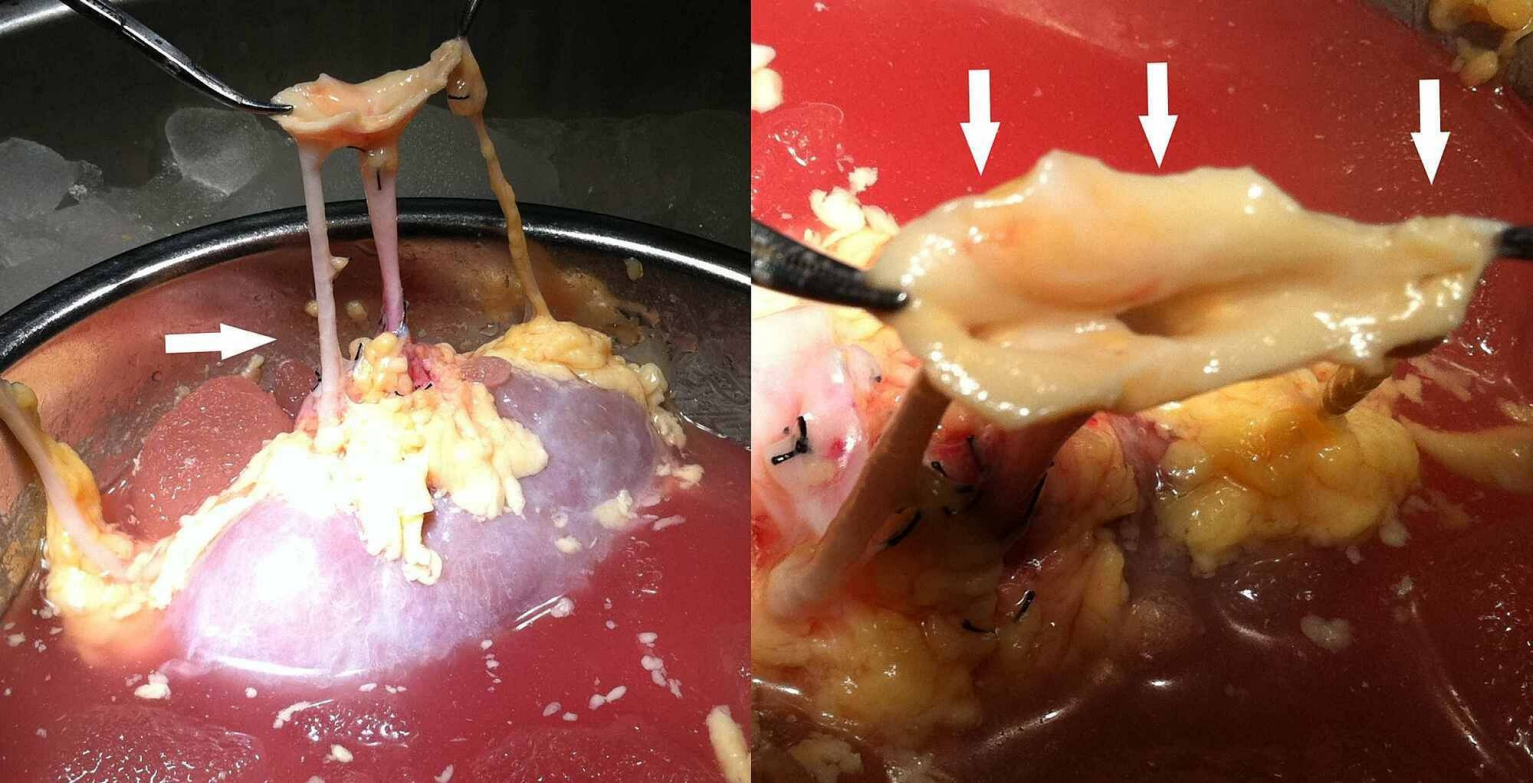
Triple renal artery found in a renal allograft from a cadaveric donor. A: Two accessory renal arteries are observed during the back-table allograft preparation. B: The three renal arteries arose from the abdominal aorta with a common aortic patch (Carrel patch).

A 42-year-old healthy male with traumatic brain injury became a multiorgan donor. During the surgical procedure of heart-beating organ donation, the left kidney was found with multiple RAs. Furthermore, during the back-table allograft preparation, the left kidney was found to have three RAs: one main RA and two accessory RAs. The main RA and the accessory RAs arose from the abdominal aorta (Figure 1A). The three RAs were preserved on the common aortic patch (Carrel patch) (Figure 1B) for the cadaveric renal transplantation procedure [7]. The ureter and the renal vein were normal and without any anatomical variation.

A 58-year-old woman with polycystic kidney disease and chronic renal failure undergoing hemodialysis over the past five years presented to our renal transplantation unit as a potential transplant recipient. After the preoperative evaluation, our patient underwent heterotopic renal transplantation. Via a Rutherford-Morrison incision, the left renal allograft was placed to the right iliac fossa. The renal vein was anastomosed end-to-side to the external iliac vein; the Carrel patch was anastomosed end-to-side to the internal iliac artery; an extravesical ureteroneocystostomy (Lich-Gregoir) using a pigtail was performed [7].

The operation was uneventful and nine years postoperatively, the renal allograft recipient remains fit and healthy, with normal renal function.

## Discussion

The three phases in the development of the urinary system include the development of the pronephros, mesonephros, and metanephros. The pronephros occurs during the fourth week of development, is located far caudally in the pelvis, and opens into the cloaca. It usually degenerates by the 25th day but most of the pronephric ducts persist and are used by the mesonephros. The mesonephros is created between the 24th day and the 16th week of gestation caudal to the pronephros and is supplied by temporary aortic branches. The metanephros migrates from the pelvic to the lumbar region in the retroperitoneal area between the sixth and ninth week until it contacts the adrenal glands [8].

During this migration, the metanephros is supplied from the iliac arteries and later from the branches of the dorsal aorta. The 5-9 mesonephric arteries formate the rete arteriosum urogenitale in the area between the mesonephros, the metanephros, and the reproductive gland. This network connects the vessels of the metanephros with the mesonephric arteries and the abdominal aorta. When one of the mesonephric arteries persists, it becomes the permanent main RA. If more than one mesonephric artery persists during the transition period from mesonephros to metanephros, this results in the formation of multiple RAs [5].

Several cases of multiple renal arteries have been reported in the literature. Young et al. mentioned for the first time in 1903 four cases with abnormalities in RAs discovered during cadaver dissection. In the first case, the right RAs were four in number, three of which arose close together from the ventral aspect of the abdominal aorta while the left RAs were five in number. In the second case, both kidneys were supplied from four RAs. In the third case, bilateral three RAs were noticed and in the fourth case, the right kidney received three RAs and the left kidney received two RAs [9].

Bordei et al. described a case of a female fetal cadaver of age 30 weeks, in whom multiple RAs were observed: triple in the right and double in the left kidney. All the arteries were hilar and originated from the lateral space of the abdominal aorta [10]. Tallapaneni et al. presented a case of dissection of an elderly male cadaver, which revealed a left kidney supplied by triple RA and double accessory RAs along with the main RA [11].

Saldarriaga et al., in their cross-sectional study, investigated the frequency of additional RAs and their morphological expression in the population of Colombia. Totally, 196 renal samples were examined from autopsies. The injection-corrosion technique was used. The researchers concluded that almost a third of the Colombian population presents one additional RA and about 3% have two additional RAs [12].

Pusztai et al. reported a case of a 57-year-old male who presented with four left RAs, one main RA, and three additional RAs, discovered on multidetector computed tomography (MDCT) angiography, used to investigate the vascular system of the lower limbs [13]. Wróbel et al. described the arterial supply of a human kidney from a 75-year-old woman, a volunteer organ donor. The kidney was analyzed with contrast-enhanced computed tomography (CT) and corrosion casting was used to reveal the kidney's angioarchitecture. In the left kidney, four RAs, each originating directly from the abdominal aorta, were noticed [14].

Pestemalci et al. reported the presence of bilateral triple RAs, found on routine dissection of a male cadaver. On the right side, one additional RA originated from the abdominal aorta and one other originated from the right common iliac artery. On the left side, both additional RAs originated from the abdominal aorta [15]. Bayazit et al. reported a 48-year-old male with increasing symptoms of intermittent claudication in both lower extremities. Angiography revealed a total occlusion of the left common iliac artery, 50% long segment narrowing of the right external iliac artery, and three arteries of the right kidney [1]. Tuteja et al. recently reported a case of five RAs found in a living kidney donor during preparation for transplantation to a nine-year-old girl with a single dysplastic kidney and chronic kidney disease. This anatomical variation led to the deferral of transplantation to the young girl [4]. Zăhoi et al. described a case of right renal ectopia with malrotation and bilateral triple RAs, all originating from the abdominal aorta, on CT angiography in a 64-year-old male patient [16].

Rossi et al. reported a case of a 23-year-old woman who was examined with multidetector computed tomography (MDCT) angiography as a potential living donor for renal transplantation. Images of the kidney obtained with the MDCT angiography revealed two normal kidneys with the presence of four left RAs and three right RAs arising from the abdominal aorta [2]. Koplay et al. presented a 36-year-old male patient who was admitted as a potential living donor for renal transplantation. An MDCT angiography was performed and clearly revealed the presence of three right and two left RAs arising from the abdominal aorta. Additionally, the accessory RA arose from the inferior mesenteric artery on the left side and from the common iliac artery on the right side [17].

Miclăuş et al., in 2012, reported a rare case of a 58-year-old male with eight RAs (bilateral four) discovered by routine MDCT angiography. All eight RAs originated from the abdominal aorta [18]. Miclăuş et al., in 2014, described an unusual case of a 63-year-old male who had seven RAs (three right and four left), discovered incidentally on MDCT angiography, which was used to examine peripheral vascular disease of the lower limbs [5].

Hirai et al. reported a rare case of multiple RAs in a 78-year-old female cadaver undergoing routine dissection. The characteristic findings in the cadaver included the presence of four right and four left RAs with one common trunk (a total of nine renal arteries) [19].

Knowledge of arterial abnormalities is essential in order to reduce the rate of accidental injuries of accessory arteries during surgeries involving the kidneys, especially renal transplantation. Transplantation of kidneys with multiple arteries is technically a more demanding approach in comparison with kidney grafts with a single artery. This might pose the recipients at high risk of vascular complications due to kinking of the vessels and consequent thrombosis. The more time is needed for vascular anastomoses directly results in elongation of the warm ischemia time, which is considered as a negative factor for an ischemic insult to the graft. The elongation of warm ischemia also promotes delayed graft function occurrence. For improvement of the overall kidney transplantation success, this negative factor must be eliminated [20].

## Conclusions

This is an unusual case of a triple RA in a cadaveric kidney donor. Although double RA consists of a common anatomical variation; three or more arteries in a single kidney is less common. Kidneys with multiple arteries are more technically challenging to transplant and are associated with an increased risk of vascular complications. The risk of such complications may be greater in childhood when blood vessels are smaller. Thus, knowledge of this anatomical variation can be extremely beneficial, as it can better prepare physicians for the aforementioned surgical procedures.
